# Intestinal Dysbiosis in Patients with Histamine Intolerance

**DOI:** 10.3390/nu14091774

**Published:** 2022-04-23

**Authors:** Sònia Sánchez-Pérez, Oriol Comas-Basté, Adriana Duelo, M. Teresa Veciana-Nogués, Mercedes Berlanga, M. Luz Latorre-Moratalla, M. Carmen Vidal-Carou

**Affiliations:** 1 Departament de Nutrició, Ciències de l’Alimentació i Gastronomía, Facultat de Farmàcia i Ciències de l’Alimentació, Campus de l’Alimentació de Torribera, Universitat de Barcelona (UB), Av. Prat de la Riba 171, 08921 Santa Coloma de Gramenet, Spain; soniasanchezperez@ub.edu (S.S.-P.); oriolcomas@ub.edu (O.C.-B.); aduelo@ub.edu (A.D.); veciana@ub.edu (M.T.V.-N.); mariluzlatorre@ub.edu (M.L.L.-M.); 2 Institut de Recerca en Nutrició i Seguretat Alimentària (INSA·UB), Universitat de Barcelona (UB), Av. Prat de la Riba 171, 08921 Santa Coloma de Gramenet, Spain; 3 Xarxa d’Innovació Alimentària (XIA), C/Baldiri Reixac 4, 08028 Barcelona, Spain; 4 Departament de Biologia, Sanitat i Mediambient, Secció de Microbiologia, Facultat de Farmàcia i Ciències de l’Alimentació, Universitat de Barcelona (UB), Av. Joan XXIII 27-31, 08028 Barcelona, Spain; mberlanga@ub.edu

**Keywords:** histamine, histamine intolerance, gut microbiota, intestinal dysbiosis, histamine-secreting bacteria, diamine oxidase (DAO) enzyme

## Abstract

An underlying cause of histamine intolerance is diamine oxidase (DAO) deficiency, which leads to defective homeostasis and a higher systemic absorption of histamine. Impaired DAO activity may have a genetic, pharmacological or pathological origin. A recent proposal also suggests it can arise from an alteration in the gut microbiota, although only one study has explored this hypothesis to date. A greater abundance of histamine-secreting bacteria in the gut could lead to the development of histamine intolerance. Thus, the aim of this study was to characterize the composition of the intestinal microbiota of patients with histamine intolerance symptoms and compare it with that of healthy individuals. The study was performed by sequencing bacterial 16S rRNA genes (V3-V4 region) and analyzing the data using the EzBioCloud Database. Dysbiosis of the gut microbiota was observed in the histamine intolerance group who, in comparison with the healthy individuals, had a significantly lower proportion of *Prevotellaceae*, *Ruminococcus*, *Faecalibacterium* and *Faecablibacterium prausnitzii*, which are bacteria related to gut health. They also had a significantly higher abundance of histamine-secreting bacteria, including the genera *Staphylococcus* and *Proteus*, several unidentified genera belonging to the family *Enterobacteriaceae* and the species *Clostridium perfringens* and *Enterococcus faecalis*. A greater abundance of histaminogenic bacteria would favor the accumulation of high levels of histamine in the gut, its subsequent absorption in plasma and the appearance of adverse effects, even in individuals without DAO deficiency.

## 1. Introduction

In the last several years, there has been growing interest in characterizing the gut microbiota, both in healthy and unhealthy individuals. It is well known that the eubiotic gut microbiota has an impact on human health and well-being [1]. Although its composition is age-related and becomes more stable in adulthood, it can be altered by a wide range of factors, such as dietary habits, lifestyle, stress, antibiotic use and diseases [1,2]. While the connection between the gut microbiota and certain noncommunicable diseases, such as obesity, diabetes, cancer, gastrointestinal and neurological disorders, is being extensively studied, its role in food intolerance, including that of histamine, is still under explored [3,4,5,6,7,8,9].

Histamine intolerance is an adverse reaction to dietary histamine that appears in susceptible individuals [10,11]. This disorder is mainly provoked by a deficiency in the key enzyme responsible for histamine degradation at the intestinal level, diamine oxidase (DAO), which leads to higher absorption [12,13,14]. The accumulation of histamine in plasma can affect numerous organs and tissues due to the wide distribution of the four histamine receptors in the organism, resulting in a plethora of gastrointestinal and extra-intestinal symptoms (i.e., dermatological, respiratory, neurological and hemodynamic complaints). According to the retrospective study by Schnedl et al. (2019), the most common symptoms in histamine-intolerant patients are gastrointestinal in nature, above all abdominal distension, postprandial fullness, diarrhea, abdominal pain and constipation [15].

DAO deficiency may have a genetic origin and has been associated with single-nucleotide polymorphisms encoding a protein with reduced histamine degradation capacity [16,17]. On the other hand, impaired DAO activity can also be temporary and reversible, arising as a side effect of some widely used pharmacological drugs, such as clavulanic acid or acetylcysteine, or a secondary symptom of gastrointestinal disorders [18]. In fact, evidence supporting an intestinal origin of histamine intolerance is growing [3]. A group of Austrian researchers found that the mucosal damage caused by gastroenteritis, irritable bowel syndrome (IBS), short bowel syndrome or gastrointestinal surgery led to a concomitant decrease in DAO and lactase activities [19]. Moreover, recent studies have suggested that reduced DAO activity may be linked to nonceliac gluten sensitivity [20,21,22]. Another cause of DAO deficiency could be an alteration in the composition of the gut microbiota, although to date only one study has explored this hypothesis [23]. Schink et al. (2018) reported that the intestinal dysbiosis in patients diagnosed with histamine intolerance could contribute to mucosal inflammation, a condition that impairs DAO activity [23]. Additionally, the fact that a range of bacterial strains in the human gut are able to produce (*Enterococcus faecalis*, *Bifidobacterium pseudocatenulatum, Lactobacillus gasseri, Escherichia coli, Morganella morganii* and *Proteus mirabillis*) and degrade (*Escherichia coli* and *Klebsiella pneumoniae*) histamine suggests that dysbiosis could influence histamine levels in the intestine [24]. In this context, the aim of this work was to characterize the composition of the gut microbiota of patients with symptoms of histamine intolerance and compare it with that of healthy individuals.

## 2. Materials and Methods

### 2.1. Study Design

The study was carried out with 26 volunteers, including 12 patients diagnosed with histamine intolerance (HIT group), who were recruited from a nutrition and dietetic centre specialized in the dietary management of DAO deficiency (DAO Deficiency Clinical Institute, Barcelona, Spain). The inclusion criteria for the histamine-intolerant patients were as follows: age between 18 and 65 years; diagnosis of histamine intolerance based on two or more symptoms described by Maintz and Novak (2007) [25]; and negative results for food allergen-specific IgE. The exclusion criteria were pregnancy, lactation, having started a low-histamine diet and having taken antibiotics and/or probiotics the month before the study. The 14 healthy individuals in the control group, who were free of histamine intolerance symptoms, were recruited from the Food and Nutrition Campus of the University of Barcelona.

Demographic characteristics and clinical symptoms of all study participants were recorded. For the intestinal microbiota and histamine concentration analysis, walnut-sized stool samples were self-collected in sterile vials and stored at −80 °C until their analyses. For the HIT group, plasma DAO activity was also analyzed using a Radio Extraction Assay (REA) according to the manufacturer’s instructions (Sciotec Diagnostic Technologies, Tulln, Austria).

All participants were informed in detail about the aim and procedure of the study and gave their written informed consent prior to study inclusion. The study was approved by the Bioethics Committee of the University of Barcelona (IRB00003099).

### 2.2. Intestinal Microbiota and Histamine Concentration Analysis in Stool Samples

Bacterial DNA was isolated from stool samples using a QIAamp Power Fecal Pro DNA kit (QIAGEN, Germantown, MD, USA) following the manufacturer’s instructions. DNA concentration was measured by BioDrop µLite (Biotech, Madrid, Spain). To analyze the composition of the gut microbiota, sequencing of the V3-V4 region of bacterial 16S rRNA was performed on the Illumina MiSeq platform by the Genomic and Bioinformatic Service of the Autonomous University of Barcelona. Then, bioinformatics analysis of the microbiota composition was performed with EzBiocloud Database (ChunLab, Inc., Seoul, Korea). For the 16S rRNA amplicons, sequence data were deposited on the NCBI database by the Bioproject PRJNA811749.

Stool histamine was determined by a competitive enzyme linked immunoassay using the Histamine ELISA kit from Immunodiagnostik AG (Bensheim, Germany) according to the instructions provided by the manufacturer.

### 2.3. Statistical Analysis

Statistical analyses of participant characteristics and the histamine concentrations in stool samples were performed using the IBM SPSS Statistics 25.0 statistical software package (IBM Corporation, Armonk, NY, USA), applying Student’s t or Mann–Whitney tests after the Kolmogorov–Smirnov test for normal distribution. Differences in the microbiota composition between groups were analyzed by the Kruskal–Wallis test for non-parametric data. Alpha diversity was measured using the Shannon index and Simpson’s index, and, for beta diversity, Bray–Curtis dissimilarity analysis was performed and visualized using principal coordinates analysis (PCoA). *p*-values of *p* < 0.05 were considered statistically significant.

## 3. Results and Discussion

### 3.1. Participant Characteristics

All participants in the HIT group were female and aged between 21 and 65 years (mean 40.4 ± 12.4). In the control group, the volunteers were 71.4% female and 24–55 years old (40.4 ± 12.4), and no significant differences were observed between the sexes in any of the study parameters (Table 1). 

The symptoms described by histamine-intolerant patients are summarized in Table 2. Gastrointestinal and neurological disorders were reported by 83% of patients with histamine intolerance, followed by dermatological (50%) and respiratory complaints (33%). The mean number of symptoms per patient was 4.3, although it was striking that two patients reported 7 and 8 symptoms, respectively. Overall, the most frequently reported symptoms were bloating and headache, followed by flatulence; diarrhoea; heart burn; and abdominal, muscular and articular pain. These were also the most common symptoms in histamine-intolerant patients identified by Schendl et al. (2019) in a cohort of 133 individuals [15]. It is worth mentioning that approximately half of the patients in the present study were underweight, with body mass index (BMI) values below 18.5.

DAO plasmatic activity was deficient in 10 out of the 12 patients with symptoms of histamine intolerance (<10 U/mL). Although DAO plasmatic activity has been proposed as a potential marker of histamine intolerance, its reported prevalence varies greatly (values ranging from 8% to 88%), depending on the study and the symptoms [26]. Discrepancies in the data could be explained by the variable etiology of DAO deficiency, which may be genetic in origin, a secondary symptom of gastrointestinal pathologies, or arise from the consumption of DAO-inhibitor drugs.

### 3.2. Intestinal Microbiota Composition and Stool Histamine Concentration

**Figure 1:**
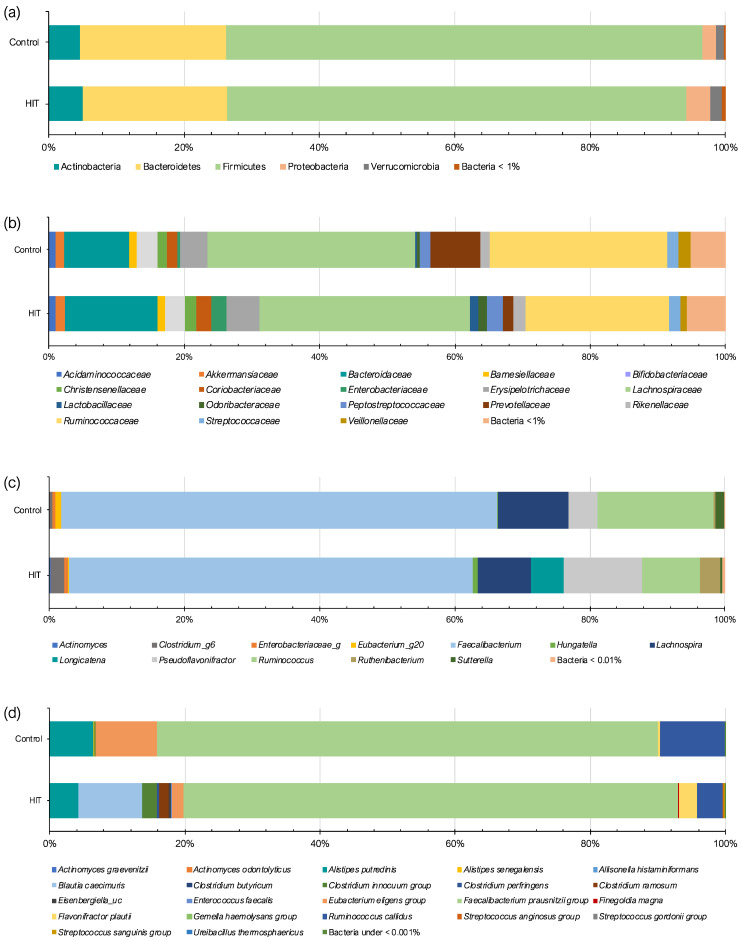
Relative abundance (%) of bacteria at the level of (**a**) phylum, (**b**) family, (**c**) genus and (**d**) species in control and histamine intolerance (HIT) groups. The genera and species are only represented if differences between the study groups were significant.

The intestinal microbiota of HIT and control groups was analyzed and compared in terms of phylum, family, genus and species. The two study groups shared a similar profile of phyla (Figure 1a), with Firmicutes and Bacteroidetes being the most dominant (approximately 90% of the total gut microbiota). Although without statistical significance, the HIT group showed a slightly higher relative abundance of the phylum Proteobacteria (3.52%) in comparison with the control group (1.88%). Similarly, Schink et al. (2018) reported higher levels of Proteobacteria in patients with histamine intolerance symptoms. According to this work, the intestinal overgrowth with Proteobacteria could result in a low-grade intestinal inflammation that could lead to epithelial dysfunction. High proportions of this phylum have also been found in patients with different intestinal disorders, such as Crohn’s disease, ulcerative colitis, colorectal cancer and IBS [27,28,29,30]. Intestinal inflammation may increase the amount of oxygen available in the intestinal lumen, resulting in a shift from obligate anaerobic bacteria towards facultative anaerobic bacteria, such as Proteobacteria. Consequently, an increase in its abundance has been postulated as a hallmark of dysbiosis [31,32].

Regarding bacterial families, *Lachnospiraceae* (Firmicutes), *Ruminococcaceae* (Firmicutes) and *Bacteroidaceae* (Bacteroidetes) represented more than 50% in both the control and HIT groups (Figure 1b). Statistically significant differences between groups were observed in four bacterial families (*p* < 0.05) (Table 3). For example, a lower abundance of *Prevotellaceae* (Bacteroidetes) was found in the HIT group. An under-representation of several members of this family group may indicate reduced mucin synthesis, which has been associated with increased gut permeability [33]. Schink et al. (2018) reported higher mean values of a marker of intestinal permeability (zonulin) in histamine intolerance patients in comparison with those recommended for the healthy population, suggesting a mild alteration of gut permeability in these patients [23]. According to these authors, an increased gut permeability facilitates the penetration of microbial metabolites, such as histamine, and, in turn, could lead to histamine-associated symptoms. Additionally, bacteria belonging to the *Prevotellaceae* family have been associated with a range of functions in the organism, such as interaction with the immune system and the synthesis of thiamine, folate and short-chain fatty acids [33].

All the genera and species identified in the microbiome of both study groups are shown in the Supplementary Material (Supplementary Tables S1 and S2). Overall, statistically significant differences were found in the relative abundance of 21 genera and 30 species between control and HIT groups (*p* < 0.05) (Figure 1c,d). In their study with histamine-intolerant patients, Schink et al. (2018) reported significant differences in five bacterial genera, including only those with an abundance greater than 0.01% [23]. Applying the same criterion, in the present study, more differences were identified at the genus level (up to nine).

**Figure 2:**
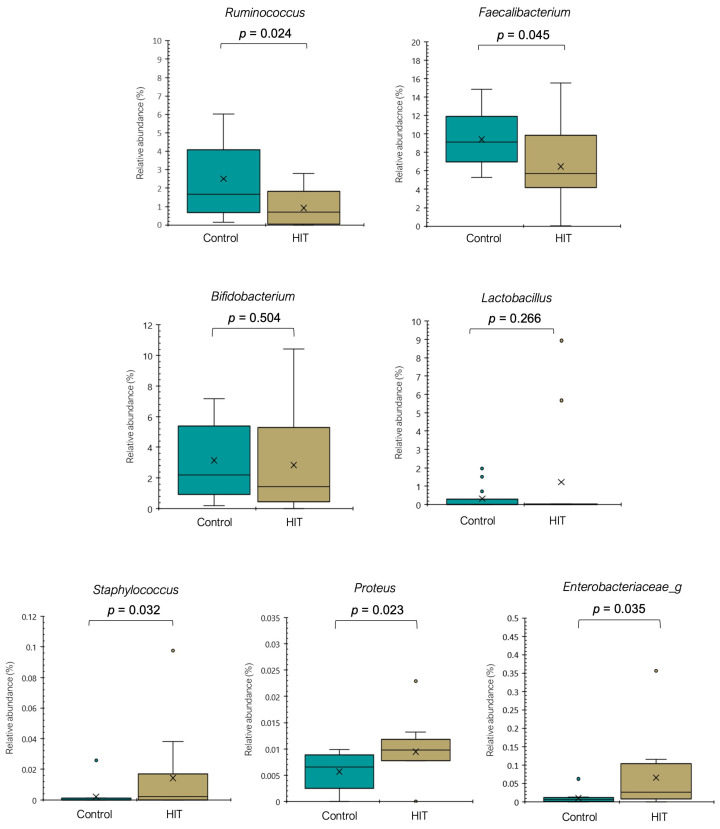
Relative abundance (%) of different genera in the control and HIT groups. Mean values are represented with an × and values statistically considered as outliers (atypical values) are plotted as circles.

**Figure 3:**
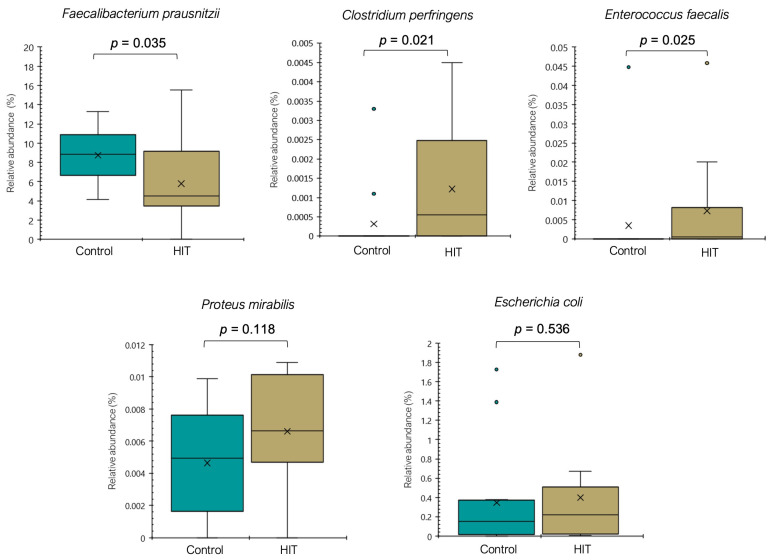
Relative abundance (%) of different species in the control and HIT groups. Mean values are represented with an × and values statistically considered as outliers (atypical values) are plotted as circles.

Although the relative abundance of *Ruminococcus* in the control group was highly variable, it was statistically significantly lower in individuals with histamine intolerance (*p* < 0.05) (Figure 2). *Ruminococcus* is thought to play a role in maintaining a healthy human gut [34]. Members of this genus can degrade complex polysaccharides into a variety of simple sugars, making them more available for the epithelium cells of the large intestine [34,35]. The relative abundance of the genus *Faecalibacterium* (Figure 2), especially the species *Faecalibacterium prausnitzii* (Figure 3), was also significantly lower in the HIT group (*p* < 0.05). Proposed as a marker of gut health, *F. prausnitzii* is one of the most prevalent and abundant producers of butyrate in the human gut, a short-chain fatty acid that represents the main energy source for colonocytes, and it displays protective properties against colorectal cancer and inflammatory bowel diseases [36,37,38]. Regarding *Bifidobacterium* and *Lactobacillus* (Figure 2), two other bacterial genera frequently associated with good intestinal health, no significant differences were found between the two groups. Only two species displayed a lower mean relative abundance in the HIT group (*Bifidobacterium adolescentis*, *p* = 0.034 and *Lactobacillus rogosae*, *p* = 0.017) (Supplementary Table S2).

Conversely, the genera *Staphylococcus* and *Proteus* were significantly more abundant in the HIT group (*p* < 0.05), with mean values 7- and 1.8-fold higher than in the control group, respectively (Figure 2). Several bacteria from these genera have shown an important capacity to form histamine [39,40,41]. Moreover, members of the family *Enterobacteriaceae*, known to be among the most prolific histamine-producing bacteria, were also significantly more abundant in the HIT group (Figure 2) [39,40,41], although they could not be identified at the genus level. It should be mentioned that the ability to form histamine is reported to be strain-dependent [42].

To date, studies on histamine-producing bacteria have been mainly focused on strains isolated from food samples. However, the histaminogenic capacity of the gut microbiota has been studied only recently, and data are still limited [32,43,44]. A systematic in silico search published in 2021 identified 117 species with a putative histamine-secreting capacity within the human gut microbiome [32], many of them belonging to genera extensively reported as histaminogenic, such as *Morganella*, *Lactobacillus*, *Staphylococcus*, *Photobacterium* and *Clostridium* [24,41,42]. For example, according to Mou et al. (2021), *Clostridium perfringens* is one of the species most frequently associated with the enzyme histidine decarboxylase, regardless of strain [32]. In the present study, the occurrence of *C*. *perfringens* (Figure 3), a bacterium responsible for several gastrointestinal disorders, was more frequently identified in the HIT group and only in two healthy individuals. Similarly, the abundance of *Enterococcus faecalis*, *Proteus mirabilis* and *Escherichia coli* tended to be higher in the HIT group (Figure 3). These species were isolated from the human gut by Pugin et al. (2017) and identified as producers of histamine as well as other biogenic amines, such as putrescine, cadaverine and tyramine [24].

It has been suggested that histamine secreted by the gut microbiota could have an impact on the health or disease status of the host. In the present study, the higher abundance of intestinal histaminogenic bacteria found in histamine-intolerant patients could have resulted in an excess accumulation and systemic absorption of histamine. Notably, patients with histamine intolerance frequently suffer from DAO deficiency, which could also enhance the toxicity of intestinal histamine. Additionally, an excess of histamine could negatively affect the inflammatory state of the intestinal mucosa. High amounts of histamine secreted by gut bacteria were linked to a proinflammatory response and signs of deteriorating health in murine specimens [43,45,46]. Mishima et al. (2020) suggested that intestinal dysbiosis, involving an over-representation of histamine-secreting bacteria and higher intestinal histamine levels, was potentially associated with the development and aggravation of IBS [47]. After analyzing 2451 stool metagenomes, Mou et al. (2021) also found that putative histamine-secreting bacteria were significantly enriched in patients with ulcerative colitis and Crohn’s disease [32]. According to these authors, the enrichment of histamine-secreting species in IBD patients was not attributed to a single taxon but highly dependent to the cohort characteristics. The involved bacterial taxons included Actinobacteriota, Firmicutes, Proteobacteria and Bacteroidiota, depending on the study; some of them were also found to be increased in the HIT patients from the current study [32]. Another study observed a higher abundance of histamine-producing bacteria in adults diagnosed with asthma [48]. These previous studies, together with the current results, support the potential association between histamine-secreting bacteria and the inflammatory status occurring in this kind of disorder. In contrast, some studies, both in vitro and in murine models, have demonstrated that intestinal histamine exerts immunomodulatory effects by suppressing the production of proinflammatory interleukines [49,50]. 

**Figure 4:**
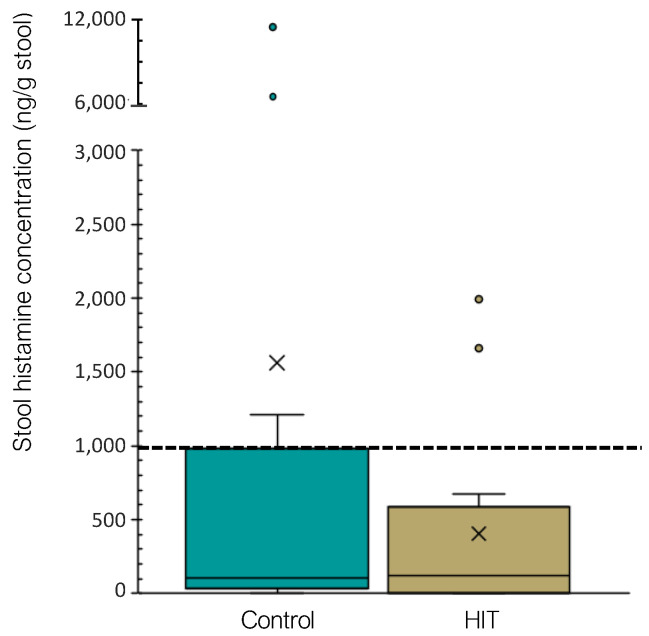
Occurrence of histamine (ng/g stool) in fecal samples of control and HIT groups. Samples above the dotted line are above the normal range (<959 ng/g stool). Mean values are represented with an × and values statistically considered as outliers (atypical values) are plotted as circles.

Concerning the histamine concentration in stools, no significant differences were found between study groups (*p* = 0.681). As shown in Figure 4, the majority of both healthy and histamine-intolerant individuals (71% and 92%, respectively) displayed fecal histamine levels within the normal range (<959 ng/g stool). The obtained results are in accordance with those of Schink et al. (2018), who also found very similar histamine levels among stool samples [23]. Therefore, the increased presence of histamine-secreting bacteria found in the HIT group was not associated with a higher histamine excretion in feces.

### 3.3. Bacterial Diversity

**Figure 5:**
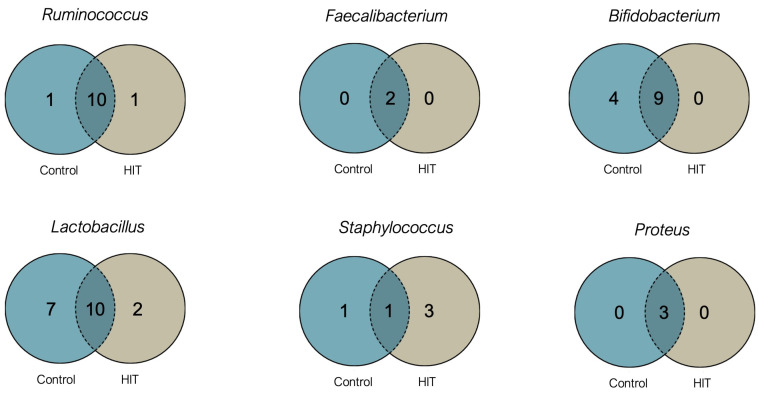
Venn diagrams of specific and shared Operational Taxonomic Units (OTUs) detected for *Ruminococcus*, *Faecalibacterium*, *Bifidobacterium*, *Lactobacillus*, *Staphylococcus* and *Proteus* genera in the control and HIT groups.

Bacterial species diversity was evaluated through indices of alpha diversity (Shannon and Simpson indices) and beta diversity (multidimensional scaling by PCoA and Bray–Curtis dissimilarity). Regarding alpha diversity, which is a measurement of the mean species diversity within the human gut, no significant differences were observed between the HIT and control groups for any of the evaluated indices (Shannon index, *p* = 0.411 and Simpson index, *p* = 0.681). Figure 5 shows the number of identified species belonging to the main genera that differed significantly in abundance between the two groups. Although the HIT group showed a significantly different proportion of genera with the capacity to form histamine (*Staphylococcus* and *Proteus*) and genera considered as a biomarker of a healthy gut (*Ruminococcus* and *Faecalibacterium*), these differences were not observed in terms of species number. However, a lower diversity in *Bifidobacterium* and *Lactobacillus* species was observed in the HIT group, with individuals showing only 69% and 59% of the species found in the control group, respectively (Figure 5). 

In disagreement with our results, Schink et al. (2018) found a lower alpha diversity in a group of 8 histamine-intolerant individuals in comparison with 10 healthy subjects [23]. Similar discrepancies exist in studies on other types of food intolerance or gastrointestinal disorders, some observing a reduced alpha diversity in patient groups [51,52,53] and others reporting no differences in this parameter [54,55].

**Figure 6:**
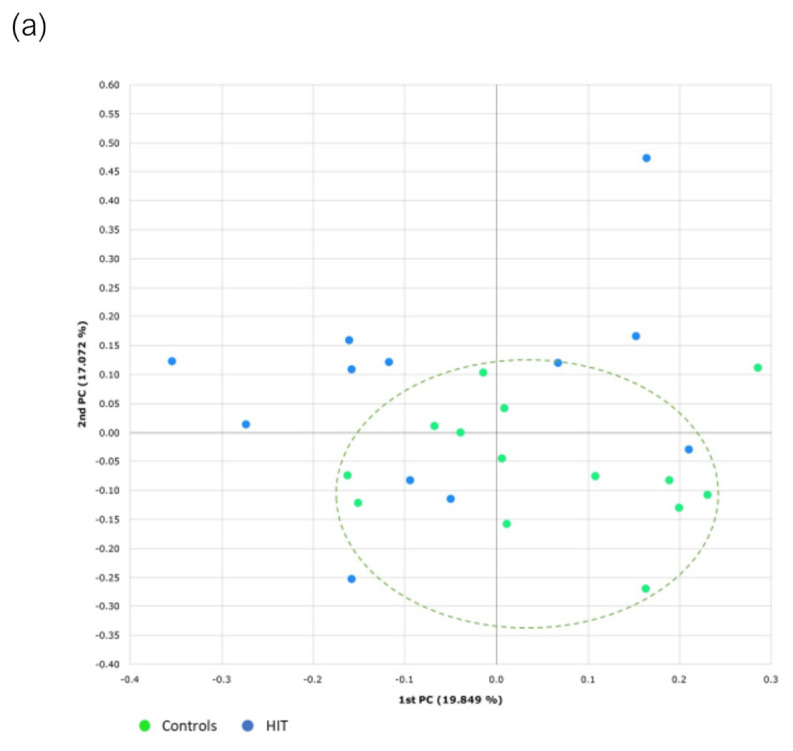

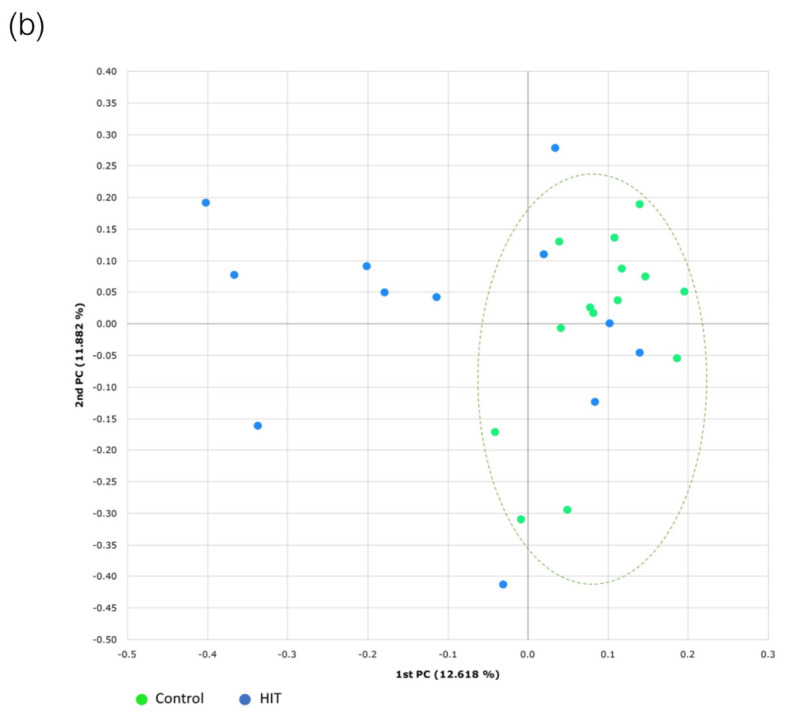
Beta diversity at the (**a**) genus and (**b**) species level determined by the Bray–Curtis index and principal coordinates analysis (PCoA). PC in both axes means “principal component”.

Beta diversity refers to the interindividual differences in the distribution pattern of genera and species. In this case, beta diversity determined by the Bray–Curtis index showed statistically significant differences between the two groups, both for genera (*p* = 0.024) and species (*p* = 0.029). As shown in Figure 6, the samples of the HIT group are more scattered compared to those of healthy individuals, which denotes a higher degree of heterogeneity in their intestinal microbiota.

## 4. Conclusions

An imbalance or dysbiosis of the gut microbiota was observed in patients with histamine intolerance in comparison with healthy individuals. In the HIT group, the relative abundance of bacteria associated with gut health, namely *Prevotellaceae*, *Ruminococcus*, *Faecalibacterium* and *Faecablibacterium prausnitzii*, was significantly lower, whereas that of histamine-secreting bacteria was significantly higher, including the genera *Staphylococcus* and *Proteus*, several unidentified genera belonging to the family *Enterobacteriaceae*, and the species *Clostridium perfringens* and *Enterococcus faecalis*. A greater abundance of histaminogenic bacteria may favor the accumulation of high levels of histamine in the gut and its subsequent absorption in plasma, which can trigger adverse health effects. The ability to degrade histamine derived from an intestinal dysbiosis would be easily overwhelmed in individuals with DAO deficiency. This dysbiosis could also provoke mucosal inflammation, a condition that impairs DAO functionality. Therefore, an over-representation of histamine-forming bacteria in the gut could be another possible origin of histamine intolerance. 

The main limitations of the present study are the small size of each sample group, the lack of male representation in the HIT group, and in some cases, the impossibility of classifying bacteria beyond the family or genus level. Moreover, the fact that approximately half of the participants from the HIT group showed a reduced BMI may also be considered as a drawback of this study as it could be another factor influencing the gut microbiota composition. These limitations should be born in mind in further studies aimed at elucidating the relationship between intestinal dysbiosis and histamine intolerance. Next steps should focus on the development of studies with a more ambitious design considering a higher number of participants to better understand until what extent an imbalance in the presence of histamine-secreting bacteria or gut health-related bacteria would be etiologically linked with the symptomatology of histamine intolerance. Moreover, it would also be of interest to assess the potential influence of the follow-up of a low-histamine diet in the intestinal microbial pattern of histamine-intolerant individuals.

## Figures and Tables

**Table 1:** Characteristics of the participants from the control and HIT groups.

Participants’ Characteristics	Groups	
	Control	HIT
*n* (%)	14 (53.9%)	12 (46.2%)
Age (average years ± SD)	40.4 ± 12.5	40.4 ± 12.4
Male [*n* (%)]	4 (28.6%)	0 (0%)
Female [*n* (%)]	10 (71.4%)	12 (100%)
Body Mass Index (BMI) [average ± SD]	23.7 ± 3.2	22.2 ± 6.0

**Table 2:** Clinical manifestations reported by the HIT group (*n* = 12).

Symptoms	Frequency (%) *
**Gastrointestinal tract**	
Bloating	75
Flatulencies	33
Abdominal pain	25
Diarrhoea	25
Heartburn	25
Constipation	17
Nausea	17
**Skin**	
Urticaria	17
Atopic skin	17
Pruritus	8
Eczema	8
**Neurologic system**	
Headache	75
Dizziness	8
**Respiratory apparatus**	
Asthma	17
Rhinitis	8
Shortness of breath	8
**Other symptoms**	
Muscular/articular pain	25
Fatigue	17
Insomnia	8

* The frequency (%) refers to the number of patients suffering these symptoms within the HIT group.

**Table 3:** Differences in the relative abundance (%) of bacterial families between the control and HIT groups. Data are presented as average ± SD.

Family	Phylum	Control	HIT	*p*-Value
Acholeplasmataceae	Tenericutes	0.001 ± 0.002	0.002 ± 0.001	0.03
Actinomycetaceae	Actinobacteria	0.012 ± 0.011	0.027 ± 0.024	0.02
Prevotellaceae	Bacteroidetes	7.422 ± 7.996	1.548 ± 2.408	0.04
Staphylococcaceae	Firmicutes	0.002 ± 0.007	0.014 ± 0.029	0.03

## Data Availability

For the 16S rRNA amplicons, sequence data were deposited on the NCBI database by the Bioproject PRJNA811749.

## Supplementary Materials

The following supporting information can be downloaded at: https://www.mdpi.com/article/10.3390/nu14091774/s1, Table S1: Bacterial genera obtained in the characterization of the intestinal microbiota in control and HIT groups; Table S2: Bacterial species obtained in the characterization of the intestinal microbiota in control and HIT groups.
